# Standards for MicroED

**DOI:** 10.1107/S2053229625004875

**Published:** 2025-06-20

**Authors:** Johan Unge, Brent L. Nannenga, Allen G. Oliver, Tamir Gonen

**Affiliations:** a Department of Chemistry, Umeå University, 901 87 Umeå, Sweden; b Chemical Engineering School for Engineering of Matter Transport and Energy Arizona State University,Tempe AZ USA; c Biodesign Center for Applied Structural Discovery Biodesign Institute Arizona State University,Tempe AZ USA; d Molecular Structure Facility, 149 Stepan Chemistry Hall, Department of Chemistry and Biochemistry, University of Notre Dame, Notre Dame, IN 46556, USA; e Howard Hughes Medical Institute University of California Los Angeles Los Angeles 90095 USA; f Department of Physiology University of California Los Angeles Los Angeles 90095 USA; g Department of Biological Chemistry University of California Los Angeles Los Angeles 90095 USA

**Keywords:** MicroED, electron diffraction, quality indicator, best practices, cryoEM, Gold standards

## Abstract

As the electron diffraction technique MicroED gains momentum and is in­creasingly embraced by researchers in both academia and industry, we have the opportunity to familiarize ourselves with the characteristics of MicroED data and results. The number of refined structures and their associated data is steadily growing and becoming more accessible to the scientific community, offering valuable insights into the significance and quality of MicroED-derived structures. Additionally, the growing body of experience is helping to identify best practices for the technique. In this summary, we highlight key lessons learned from these data and propose gold standards for the community to consider adopting.

## Abstract 2

With an increasing number of refined MicroED structures available to the community, lessons can be learned of their profiles and quality, as well as of best practices.

## Introduction

Over 100 years of crystallography started when Max von Laue with colleagues demonstrated that matter could diffract X-rays using a copper sulfate crystal in 1912. The beginnings of the development of an electron microscope started decades later. Louis de Broglie proposed in 1924 that electrons also have wave properties, such as wavelength and frequency, and this was quickly followed by confirmation of electron dif­frac­tion by Clinton Davisson and Lester Germer. An early application of the electron microscope was presented by Max Knoll and Ernst Ruska in 1931, and electron crystallography could be considered as starting with the first crystal structure analysis using electron diffraction (ED) presented in 1949 by Pinsker and Vainshtein (Vainshtein & Pinsker, 1949 ▸). How­ever, after this initial progress, the field of electron crystallography was quickly set back by the difficulties in inter­preting the diffraction patterns and correlating them to the structure of the mol­ecules in the crystals. Cowley soon found that the two-beam theory was not sufficient to explain the electron diffraction patterns, and a dynamic scattering theory was required for electron diffraction data for heavier atoms (Cowley, 1953 ▸), such as lead carbonate (Cowley, 1956 ▸). Later, it was argued that simulations of dynamical diffraction showed that phasing could be realistically possible (Dorset *et al.*, 1979 ▸). Cowley also realized that by using a smaller beam and thinner samples, dynamical scattering could be minimized. Early approaches to macromolecular electron crystallography typically involved still diffraction images taken at several fixed tilt angles (typically 0, 20, 45, and 60°). For radiation-hardy materials, rather than proteins, it was found that solving a structure *ab initio* was possible with precession electron diffraction, rather than still images, which to some extent averaged out effects of dynamical scattering (Vincent & Midgley, 1994 ▸), and later this application for materials structure matured with additional approaches (Andrusenko *et al.*, 2015 ▸; Oleynikov *et al.*, 2007 ▸). However, these techniques require precise alignment of the crystal axis on the rotation axes and in the electron beam. This requires a significant amount of expertise, specialized equipment, and data pro­ces­sing procedures, and can be difficult for samples with larger unit cells such as macromolecular crystals. Importantly, the method requires a very high exposure, limiting its usefulness for biological samples like proteins that cannot withstand the high radiation, making precession unsuitable for these samples.

In parallel with the work in materials science, electron crys­tallography for biological samples was successful for the structure solution of 2D crystals, and provided some of the earliest structural information on membrane protein structure (Henderson & Unwin, 1975 ▸; Unwin & Henderson, 1975 ▸; Grigorieff *et al.*, 1996 ▸; Kühlbrandt *et al.*, 1994 ▸; Kimura *et al.*, 1997 ▸; Fujiyoshi, 1998 ▸; Murata *et al.*, 2000 ▸; Gonen *et al.*, 2004 ▸, 2005 ▸). The first high-resolution model of bacteriorhodopsin to 3.5 Å resolution was quickly followed by the structure of the light-harvesting chloro­phyll complex to 3.4 Å (Kühlbrandt *et al.*, 1994 ▸). However, the application of electron crystallog­raphy on thin 3D macromolecular crystals remained elusive. Early work by Dorset and Parsons (Dorset & Parsons, 1975*a* ▸, 1975*b* ▸) demonstrated that electron diffraction data from thin catalase crystals could be treated kinematically. However, full data sets from each crystal were not collected. Some of the earliest work on collecting tilt series of thin 3D crystals were performed on biological crystals (Shi *et al.*, 1998 ▸; Jeng & Chiu, 1984 ▸; Brink & Chiu, 1994 ▸). Seminal work in this area was reported by Wah Chiu and Stokes (Jeng & Chiu, 1984 ▸; Shi *et al.*, 1998 ▸), predating much of the work on materials, and out­lined procedures for collecting multiple diffraction patterns from a single crystal. While this demonstrated that this approach was possible, these studies were not capable of determining structures.

It was not until 2013 that structure determination of bio­logical material from thin 3D crystals using electron diffraction became a reality, with the advent of the cryoEM method known as Microcrystal Electron Diffraction or MicroED (Shi *et al.*, 2013 ▸; Nannenga *et al.*, 2014 ▸). In MicroED, a crystal is continuously rotated in the electron microscope while electron diffraction data are collected using a fast camera as a movie. Because electrons inter­act with matter 1000× better than X-rays do, the samples for MicroED are much smaller than for X-ray. The ideal thickness of a crystal for MicroED is about 10–400 nm for the most commonly used microscopes, thus making a complete diffraction data collection possible from a crystal a billionth the size of a crystal usually encountered in X-ray crystallography. This approach solves several problems at once: (1) the effects of dynamical scattering are efficiently averaged out and reduced from the collection of the entire reflection intensity profiles during rotation enabling structure solution; (2) there is no need to orient the crystal prior to data collection, so very low exposures could be used and biological material could be inter­rogated; (3) as the experimental setup is similar to X-ray crystallography, MicroED data could be processed using the highly sophisticated data reduction software packages that were developed for X-ray crystallography. This approach meant a major step forward for electron crystallography and opened the door to many new applications and opportunities, and a wide adoption of MicroED by the scientific community (Jones *et al.*, 2018 ▸; Unge *et al.*, 2024 ▸).

Since the first structure using MicroED in 2013, 138 entries of proteins and peptides have been deposited to the Protein Data Bank (PDB) (Berman *et al.*, 2000 ▸) as of February 2024, and these include many targets that could only be determined using MicroED. These targets include, but are not limited to, drugs, peptides, natural products, macrocyclic drugs, prions, engineered enzyme variants, proteases, ion channels, viral proteins, peptidic anti­biotics, amyloid peptides, enzymes, and G-Protein Coupled Receptors (GPCRs), demonstrating the general applicability of MicroED in current structural biology. For many of these samples, the limitations in crystal growth could not be overcome (Porter *et al.*, 2022 ▸; Gillman *et al.*, 2023 ▸; Clabbers *et al.*, 2021 ▸; Xu *et al.*, 2019 ▸) and hindered structure determination for years. However, they are ideal for structural investigation using MicroED (Lin *et al.*, 2023*a* ▸, 2023*b* ▸, 2024*b* ▸; Karothu *et al.*, 2023 ▸).

The application of MicroED has also attracted a growing inter­est for small mol­ecules, complementing existing techniques with new opportunities. The possibility of using crystals down to 10 nm in thickness enables samples to be analyzed directly from dry powder (Bruhn *et al.*, 2021 ▸; Sekharan *et al.*, 2021 ▸). This circumvents the need to find conditions to grow crystals suitable for single-crystal X-ray diffraction (SCXRD), which may be difficult for reasons of flexibility, non-specific contacts, or chemical stability of the compounds developed in modern organic chemistry. For some of those mol­ecules, recrystallization turned out to be unfruitful or hindered by sample reactivity, stability, or availability. For several samples, the crystallinity is low and the ratio of grains with at least decent diffraction may be too low for a feasible workflow. The resolution to which the crystals diffract may vary between the grains less than a micrometer in size, which is still doable in the electron beam of similar width. MicroED may also complement Powder X-ray Diffraction (PXRD), where structure determination can be difficult for mixtures or larger unit cells, where the diffraction peaks overlap. Using MicroED, single-crystal lattices and phases can be separated and studied individually (Lightowler *et al.*, 2022 ▸; Yokoo *et al.*, 2024 ▸), and may include a quantitative analysis of the crystalline content of the powder (Unge *et al.*, 2024 ▸). MicroED is also the method of choice when the amount of sample is limited, for example, for natural products (Kim *et al.*, 2021*a* ▸). Its capacity for small crystals has led to the reanalysis and update of the structures of several pharmacological compounds (Lin *et al.*, 2024*a* ▸; Kim *et al.*, 2021*a* ▸, 2021*b* ▸). The Cam­bridge Structural Database (CSD; Bruno *et al.*, 2002 ▸; Groom *et al.*, 2016 ▸), directed towards small mol­ecules, contains more than 740 structures determined by electron diffraction. Of these, 270 structures state MicroED or the synonymous keyword cRED (continuous Rotation Electron Diffraction) as the data col­lection method. The remaining electron diffraction entries in the CSD are either a result of other ways to collect diffraction data in an electron microscope or not tagged in a similar way. Other methods include Precession Electron Diffraction, where the crystallographic axes tend to be aligned with the beam prior to recording diffraction data, using small movements of the beam (‘precession’). This is more suitable for inorganic material that is less sensitive to radiation damage due to the extra alignment step. Also included are data acquisition strategies similar to tomography and X-ray Free Electron Laser (XFEL) measurements using Serial Electron Diffraction (SerialED). These measurements sample multiple crystals at a fixed tilt angle to obtain a high data completeness and distribute the potential radiation damage across the specimen. Due to the conceptually easy data collection strat­e­gy and the similarity to measurements at most synchrotrons, MicroED is no longer a technique restricted to a few groups. As such it is expected to continue to grow as a technique, reaching a wider application field. Perhaps the final hurdle is availability of dedicated MicroED equipment and facilities, which are still sorely lacking for the scientific community. Once those come online we anti­cipate the exponential growth of this field of research to increase even faster.

## A brief overview of the MicroED workflow

### Sample preparation

MicroED sample preparation generally follows one of two routes depending on whether the sample is suspended in a liquid or is a dry powder containing submicron crystals. Solution-im­mersed samples, such as for macromolecular crystals or small mol­ecule crystals in suspension, need to be applied to an electron microscopy grid with a thin layer of carbon support. For protein and nucleic acid samples, it is important that the crystals do not dehydrate, which will severely affect the crystallinity and therefore the diffraction quality. For these macromolecular samples in aqueous buffers, excess solution must be blotted away before vitrification in liquid ethane. For crystalline suspensions of small mol­ecules in solvents with low volatility, the sample may also need to be blotted to remove excess solvent. If the solvent is highly volatile, the sample can be allowed to dry before loading into the microscope. Dry powder samples, on the other hand, can be applied to the grid surface directly. Grains in a powder that are too large can usually be ground to an appropriate size simply by grinding the sample between glass microscope slides or using a mortar and pestle. For crystals that are too thick for beam penetration, cryo-Focused Ion Beam (cryo-FIB) milling can be used to process the crystals to the proper thickness for MicroED data collection (Martynowycz *et al.*, 2019 ▸). Large crystals can sometimes be disintegrated to small enough crystallites using techniques frequently exercised for providing seeding fragments in a crystallization setup or simply by manually cutting the crystal to an appropriate size using dedicated tools for this purpose (Danelius *et al.*, 2022 ▸; Jones *et al.*, 2018 ▸; de la Cruz *et al.*, 2017 ▸). Each of these samples and methods may have a different grid type which may be ideal, thus it is important to test several different grid types.

### Data collection

Accurate and careful alignment of the electron beam is necessary for a successful experiment. The electron beam is easily and accurately bent and controlled using the magnetic lenses of the electron microscope. As a consequence, any aberrations or imperfections in the lenses will be directly translated to the resulting diffraction pattern on the detector, which if uncorrected can lead to distortions of the diffraction pattern. The built-in deflectors assigned to compensate for these un­intentional effects need to be properly set to ensure an evenly distributed and circular beam on the target. As the optimal portion of an electron lens is very small, the electron beam also needs to be aligned accurately onto the optical axis. With a well-aligned electron beam and a diffraction pattern of decent intensity, the processing of MicroED data is straight­for­ward using software that was originally intended for X-ray <!?up><!?tlsb><!?down>crystallographic diffraction. Small misalignments of the crystal, beam, detector, or rotation axis are always present, and the data processing software usually allows for a certain degree of tolerance to imperfections of the regularity of the diffraction pattern. However, larger deviations or distortions of the diffraction patterns can lead to errors in the estimation of the spot position or calculation of the beam profile. This can lead to incorrect integration of the diffracted intensities (Brázda *et al.*, 2022 ▸), resulting in sub­optimal data or even failure to pro­cess the data.

For successful data collection, the dose (exposure) needs to be calibrated and properly selected. For protein data collection, it is recommended to use less than 1 e^−^ Å^−2^ total dose during data collection. The camera speed needs to be optimized in relation to the rotation speed of the stage. Generally, one should aim to collect the full rotation range of 140° from each sample to maximize the attainable data. Data collection then becomes a race against radiation damage. Several reviews and protocols have been published that delve into the best practices for data collection and integration (Otwinowski & Minor, 1997 ▸; Rajashankar & Dauter, 2014 ▸; Gonen, 2013 ▸; Hattne *et al.*, 2015 ▸).

Because of preferred crystal orientation, low symmetry, and the limitations of the microscope rotation stage, sometimes the data will be less than 100% complete. These missing reflections will often lead to an empty and unsampled volume in reciprocal space, and an uneven distribution of the measured reflections known as the ‘missing cone’ (Dorset, 1999 ▸). When the missing volume of data is severe, this impedes structure determination significantly. To minimize or eliminate the missing cone one could use the suspended drop approach, as was described recently (Gillman *et al.*, 2024 ▸). When crystals do not adopt a preferred orientation, collecting data from several crystals allows data sets to be merged and for the reciprocal space to be completely sampled. For example, for both catalase and the SARS-CoV-2 Mpro, the coverage was drastically enhanced by measuring multiple crystals to reach about 95% completeness. This suggests that even with low crystallo­graphic symmetry, high completeness can be achieved if data from a number of crystals are merged. If the crystals do not possess strong preferential orientation, a larger fraction of the available reflections could be recorded for a better completeness of the merged data.

### Background scattering and noise

The background scattering in MicroED data may be an important consideration when setting up data collection. The background of the diffraction images is caused by inelastically scattered electrons from the sample, as well as scattering from the amorphous portion of the sample, which together results in diffuse scattering. In addition to the background from the electron beam, the detector will contribute to the noise of the background, which can be reduced by using newer detectors. Smaller rotation angles per frame can decrease the amount of background per image when coupled with faster detectors for electron microscopy. These detectors allow for a frame readout speed of hundreds or even thousands of frames per second and the ability to scan the rotation in greater detail allows better separation of the diffraction reflections from the background. As a result, the diffraction data is greatly im­proved in quality and resolution (Hattne *et al.*, 2023 ▸). Additionally, energy filters can remove inelastically scattered electrons and, if the microscope is equipped with the rather expensive device, can greatly reduce the amount of background in the data (Clabbers *et al.*, 2025 ▸). Using this approach, subatomic resolution was obtained even for proteins.

## A brief overview of data processing

### Integration

For processing of MicroED data from macromolecules or large unit cells, the same software that has been developed for X-ray crystallography can be generally used, including *XDS*, *MOSFLM*, *DIALS* and *HKL2000* (Kabsch, 2010*a* ▸, 2010*b* ▸; Battye *et al.*, 2011 ▸; Parkhurst *et al.*, 2016 ▸; Sheldrick, 2015 ▸; Otwinowski & Minor, 1997 ▸; Clabbers *et al.*, 2018 ▸). Small mol­ecule data can be treated in the same way using already familiar software, including the software used for macromolecules, or using software specifically developed for electron diffraction, such as *PETS2* (Palatinus *et al.*, 2019 ▸). While these programs work very well for MicroED data, there are also a few exceptions to be aware of as a result of the differences between electron and X-ray diffraction experiments. The wavelength of the electrons typically used in transmission electron microscopy (energies greater than 80 kV) are much shorter than what is used for X-ray diffraction and the detector distances are significantly longer. Additionally, the lenses of the electron microscope create aberrations, such as astigmatism, which is more tolerable in a well-aligned microscope, but the diffraction patterns may still include subtle distortions that could render the data processing more difficult.

### Scaling and merging

Frequently data from several crystals needs to be merged to increase completeness. The number of data sets required will depend on the orientation of the crystals and the crystal symmetry. The standard software for scaling X-ray data is also applicable for MicroED data. Both *POINTLESS*/*AIMLESS* (Evans & Murshudov, 2013 ▸) and *XSCALE* (Kabsch, 2010*a* ▸) have been used successfully by our research groups, while *DIALS* also supports the use of several crystals. When merging data from multiple crystals, it is important to ensure that the correlation between each crystal in the final merged data set is reasonable; adding several data sets to increase completeness, with a low correlation to the rest of the data, may ultimately result in worse overall data for structure solution and refinement.

### Phasing

For macromolecular MicroED structures, the Mol­ecular <!?up><!?tlsb><!?down>Replacement (MR) method is usually a preferred way to phase the data and is often a rapid and easy approach if there is a good model at hand. The first demonstration of MR in electron diffraction was reported as early as 2004 (Gonen *et al.*, 2004 ▸). If the structure has not been determined previously and there are not any sufficiently homologous proteins, *AlphaFold2* (Jumper *et al.*, 2021 ▸) can be used to generate search models. Recently, *Alphafold2* was used successfully to phase the MicroED data of a previously unknown protoglobin target, a variant engineered through directed evolution (Danelius *et al.*, 2022 ▸). It has also been applied to phase a protein with un­known function (Miller *et al.*, 2024 ▸). The advent of *AlphaFold2* promises to expand the applications of MR for MicroED data. In the case of high-resolution and high-quality data, fragment-based *ab initio* phasing has also been applied to both peptide (Richards *et al.*, 2021 ▸) and protein samples (Martynowycz *et al.*, 2022 ▸). For the highest resolution structure, only a generic three amino acid polyalanine model was required to initiate the phasing of the entire structure. In MR, the chances of a successful positioning of the model within the unit cell is intrinsically dependent on the resolution and completeness, but also on other quality indicators of the data, such as the signal-to-noise ratio [*I*/σ(*I*)]. With MicroED, as with X-ray data, there is not always a clear-cut indication for when a structure is correctly solved by the MR software, or what quality of data is needed for a successful structure determination. In general, a high completeness and high resolution is an advantage, and spending another few days on the data collection step to improve the data will generally save significant time for difficult cases in the end.

The structures of smaller mol­ecules, such as pharmaceutical, organic, metal–organic, or natural compounds, are often approached using *ab initio* or direct methods, which provides a structure solution without any prior assumptions. Small mol­ecule samples regularly result in atomic resolution data and, at the same time, the number of atoms is smaller and therefore well suited to direct methods. More recent advances in small mol­ecule structural solution techniques (so-called Intrinsic Phasing; Sheldrick, 2015 ▸) are also applicable. Direct methods require high-resolution data to resolve atoms (Sheldrick, 1990 ▸). The empirically found rule, called the Sheldrick 1.2 Å rule, emerged from the discovery that a structure was unlikely to be solved unless half the number of theoretically measurable reflections in the range 1.1–1.2 Å are recorded and have a signal of *F* > 4σ(*F*). Granted its importance as a practical confirmation of your data, most entries in the CSD instead use a lower threshold of about *F* > 2σ(*F*) (although variations are plenty). While lacking a correspondence between benchmarks, including as much data as possible can support the phasing of the structures better and since the Sheldrick rule was first described, the view of using a signal-to-noise measure for a resolution cutoff has also shifted. As an example, macromolecular structure determination now commonly uses either no sigma cutoff, a lower sigma cutoff, or a CC_1/2_ cutoff, which makes it more difficult to compare with older standards in general (Karplus & Diederichs, 2015 ▸). For other data-quality indicators, such as completeness and the signal-to-noise ratio of the data, the threshold for a successful structure determination is perhaps not similarly as decisive as the resolution, and the parameters are also mutually inter­de­pen­dent. For instance, for a phasing approach using direct methods with only weaker diffraction data (low signal-to-noise ratio) available, a higher overall resolution and a com­pleteness close to 100% could be advantageous. Even without exact thresholds for data-quality indicators, such as completeness or the signal-to-noise ratio, these properties are never­theless crucial for a successful *ab initio* structure determination. For phasing, the completeness is directly linked to the structural information that can be extracted from the data. A completeness close to or exceeding 80% would be the goal as it is more likely a clear structure solution can be found. In contrast, for data where too low completeness results in a large missing cone of data in one direction, the systematically missing reflections could lead to the failure of direct methods. Even if the solution is found with systematic low completeness, the atomic positions are effectively less resolved in this direction. For phasing, therefore, completeness is of foremost inter­est, and a proper scrutinizing process should preceed publishing structures subject to very low completeness. In our workflow, *SHELXT* (intrinsic phasing) is often the software of choice for structure solution, which almost immediately pro­vides the correct structure when data quality is high. As the chance of structure determination greatly increased with high completeness and with data exceeding 3–4 σ to higher resolution, the highest quality data possible should be the goal. There are other programs that can be used for structure solution, such as *SIR2014* (Burla *et al.*, 2015 ▸), *SUPERFLIP* (Palatinus & Chapuis, 2007 ▸), and *SHELXD* (Sheldrick, 2010 ▸). Although *SHELXD* was originally intended for macromolecular phasing through heavy atom or anomalous peak search, it has been used successfully in the *ab initio* phasing of small mol­ecule structures. Examples include, for instance, the macrocyclic compounds romidepsin and paritaprevir, for which data were collected to 0.8 and 0.85 Å, respectively (Danelius *et al.*, 2023 ▸), and two additional crystal forms of the Hepatitis C virus active compound paritaprevir that were solved to 0.85 and 0.95 Å, respectively (Bu *et al.*, 2024 ▸).

In addition to direct methods, the use of MR on small mol­ecule data has been reported and seems to be a promising alternative. This is particularly important for samples dif­fracting to a lower resolution required by direct methods. As many samples where MicroED is being used have already been approached using X-rays, it is often the case that MicroED is pursued as the last resort for difficult samples and diffraction is known to be compromised. The same software being used for macromolecules can in principle be used also for small mol­ecules, although in some cases the unit-cell content calculations will be off, limiting a direct application. To handle the relative rotation of flexible parts of the mol­ecules, schemes to dissect the mol­ecule in rigid parts that can be simultaneously searched for have been applied successfully (Gorelik *et al.*, 2023 ▸).

### Refinement and model building

Refinement of small mol­ecules and large mol­ecules uses the same software as for X-ray crystallography and with similar settings. *SHELXL* and *Refmac5* have been used successfully to refine small mol­ecule models. *Refmac5* (Murshudov *et al.*, 1997 ▸) and *Phenix* (Liebschner *et al.*, 2019 ▸) are used frequently for the refinement of macromolecular targets. Just like for X-ray crystallography, there is the risk of overfitting the model to the data, particularly when data only extend to low resolution or if the data have low completeness. Similar precautions apply and the *R*_free_ value can help in determining if the model is accurate or overfitted. A difference between the re­finement quality indicators *R*_work_ and *R*_free_ that is substanti­ally larger than 5% is usually an indication of a refinement pushed towards a single refinement indicator (target), and structures reporting these values should be looked at critically. As MicroED data seem to have a different noise profile, pre­sum­ably from the differences in the background and dynamical scattering as compared to X-ray, a higher level of precaution in terms of overfitting seems sensible. Small mol­ecule data typically do not have a parameter corresponding to *R*_free_ and overfitting is guarded from by considering the ratio of the number of experimental observations (reflections) to the number of variable parameters used in the refinement.

**Figure 1:**
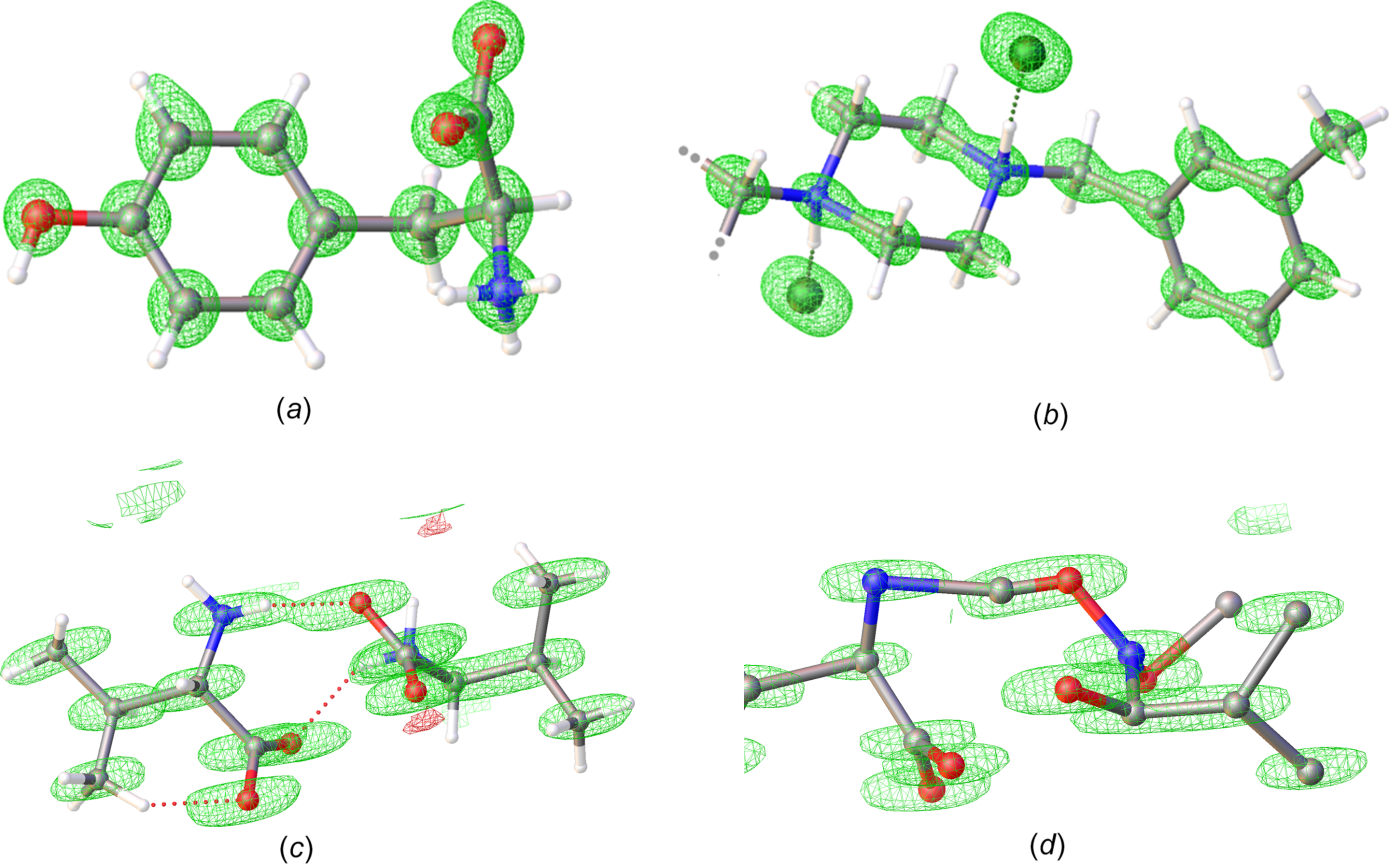
The potential maps from MicroED structures with different levels of completeness of data are presented. (*a*) The refined structure of tyrosine from a commercially purchased powder sample, using overall 96.5% complete data at 0.75 Å resolution. (*b*) The structure of the long-time used anti­histamine Meclizine using 80.7% of possible reflections at 0.96 Å. (*c*) The structure of valine using 49.4% complete data at 0.75 Å resolution. The missing data, predominantly in one direction (the ‘missing cone’), results in elongated map densities and less determined coordinates during refinement. (*d*) The output from *SHELXT* for the same data as in part (*c*), showing that the missing data causes *SHELXT* to accidentally include an extra atom in the first model despite the high nominal resolution of the data.

Extending the model with additional atoms may be difficult when the phase errors or lack of high-resolution data inhibits a more detailed map. In that case, it is recommended to support the placement of atoms during refinement with additional verification of an improved model. As a general rule and with very few exceptions, the *R*_free_ or *R*_1_ value tends to decrease with a better model. For macromolecular data, an omit map that is calculated without the region of inter­est is usually calculated to allow the calculation of a bias-free map. Before the map is calculated with the region of inter­est left out, the structure is usually subjected to simulated annealing to reset the coordinates from any shifts that resulted from bias towards the model. For small mol­ecule refinement, the missing cone can be problematic, at least for the initial rounds of refinement, as the exact positions of the atoms are less defined in the direction of the missing data. In our experience, it may be important to fix the bond lengths and angles to ideal values early in the refinement process to get better convergence of the refinement parameters. Additionally, the use of anisotropic refinement may not be appropriate with low completeness. As a rule, we generally avoid anisotropic refinement when the completeness is less than approximately 85% (see Fig. 1 ▸ for example).

## Data and refinement statistics

For evaluation, the structures from both the PDB and the CSD were organized into groups where MicroED was the main method of structure determination and groups excluding MicroED, with the vast majority being X-ray structures. The MicroED structures from the PDB that were included in our analysis span a resolution range from 0.75 to 3.4 Å. The structures extracted were included in the statistical analysis only if a minimum number of parameters being extracted was present in the CIF file, including the resolution and *R*_free_. An initial search for MicroED structures was done using ‘electron crystallography’ for the method label, which also includes structures from 2D crystallography and SerialED. The subset was further refined using both keywords and manual examination of both the PDB header and the primary publication, resulting in the PDB (MicroED) group of structures consisting of 116 structures. This group of MicroED structures was com­pared with all X-ray-generated structures in the PDB, which were extracted using the same script and the keyword ‘X-ray’, and which generated a much larger group of more than 177000 structures, here referred to as the PDB (X-ray) set. Similarly, MicroED entries were extracted from the CSD using key­words in the comment field expressing either MicroED or cRED, taking variants in upper and lower case into account. Entries where SerialED, Precession Electron Diffraction (PED), or methods using tilt series were mentioned were not used for consistency. In addition, entries were only included if a number of statistical parameters were present to support the quality of the entry, namely, highest/lowest *hkl* values, unit cell, goodness-of-fit (GooF), *R*_1_(all), *R*_1_(*I* > 2σ), number of reflections above 2σ, number of parameters, and finally the number of restraints in the refinement. This left us with 113 <!?up><!?tlsb><!?down>structures for analysis, which we denote as the CSD (MicroED) set. For the comparison group, a subset was down­loaded manually from the CSD with the same requirements as for the MicroED structures, while excluding all entries men­tioning electron diffraction, and are referred to as the CSD (Non-ED) set, consisting of 1023 entries. Entries were selected randomly based on their database ID over the entire name space, except for entries flagged as electron diffraction. For all groups, the statistics were extracted using Python scripts reading the CIF and mmCIF files, as the Python inter­face from the CCDC could not be used due to lack of access to parameters of inter­est. Below we discuss the acceptable range and gold standards for each.

### Resolution

**Figure 2:**
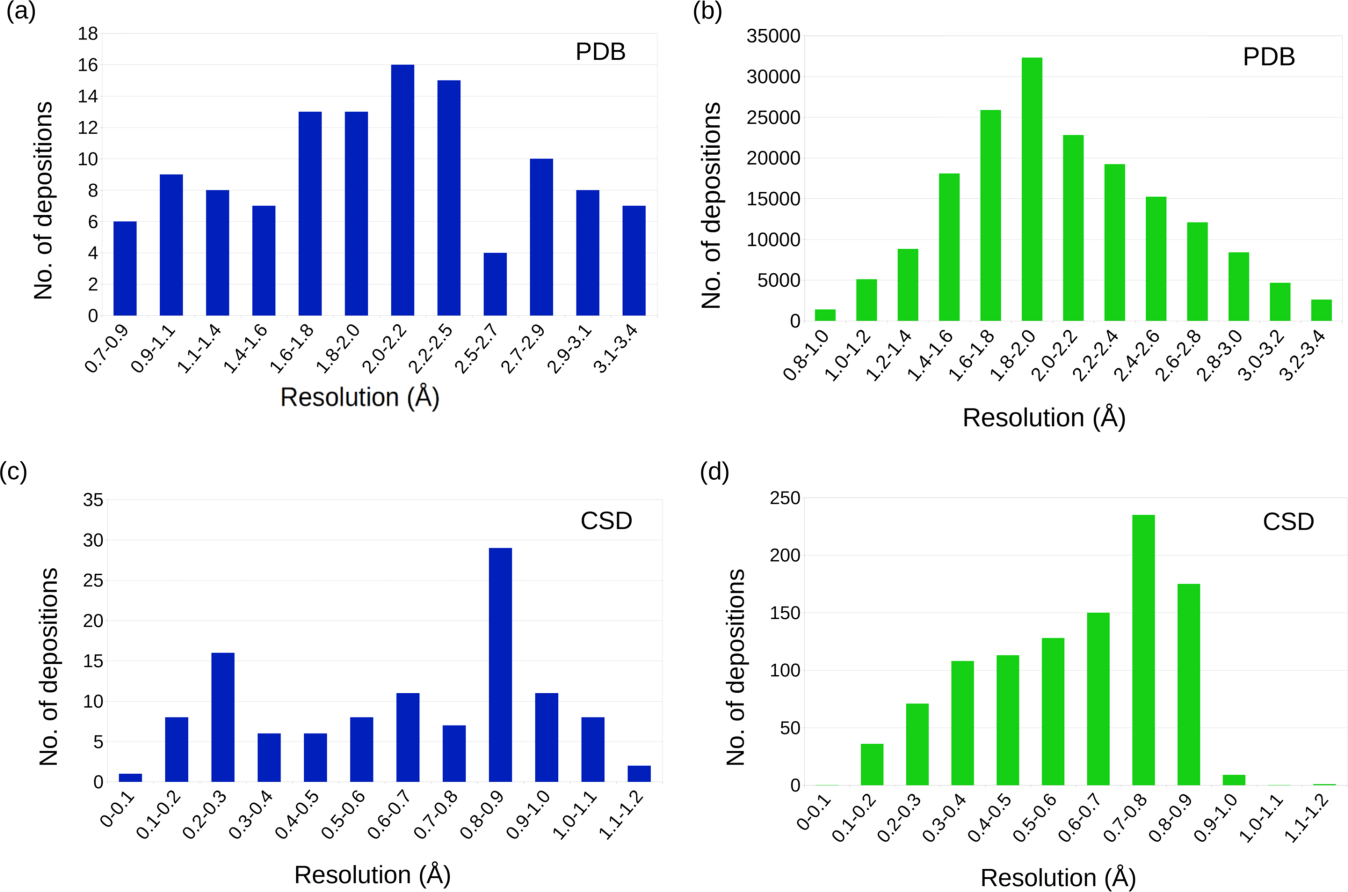
The number of structures plotted for each resolution bin for MicroED structures (blue) and X-ray structures (green). (*a*) The MicroED structures in the PDB show a widespread distribution with a maximum better than 2 Å as compared to the sharper profile for the X-ray data which is centered at 2 Å (*b*). The corresponding statistics for the MicroED structures in the CSD (*c*) are again more widespread compared to the organic Non-ED small mol­ecule structures (*d*).

In Fig. 2 ▸(*a*), the number of macromolecular MicroED de­positions is plotted as distributed over the entire resolution range. Although this represents a limited set of structures com­pared to the similar plot of the X-ray structures in the PDB [Fig. 2 ▸(*b*)], the distribution presents a peak for the number of deposited structures at around 2 Å. At the same time, the distribution for the MicroED structures is more widespread. This may be a result of the nature of the MicroED analyzed structures (see *Conclusion*) or variations in the workflow. Inter­estingly, the peak for the most frequent resolution range for MicroED structures is also found at 2 Å. This confirms the expectation that the increased inter­action between the electron beam and the crystals would counterweight the smaller crystal volume. In other words, despite the order of magnitude smaller crystals utilized in MicroED, the final resolution can be expected to be similar for MicroED and X-ray data. The resolution is typically not stated explicitly in the CIF files from the CSD and was therefore calculated from the single reflection accepted of highest resolution using the common cutoff at *I* > 2σ(*I*) for the purpose of comparison. The mean resolutions for all structures analyzed are again very similar between the groups; the CSD (Non-ED) group dis­plays a resolution of 0.60 Å, compared to 0.64 Å for the CSD (MicroED) group [Table 1 ▸, and Figs. 2 ▸(*c*) and 2(*d*)]. The dis­tribution of the resolution in the CSD is similarly more widespread for the MicroED group compared to the CSD (Non-ED) group, which has a sharp fall-off at a resolution of about 1 Å. As a side note, the fact that MicroED contains several structures at a resolution lower than 1 Å can be con­sidered as an increased demand for phase-determining techniques, in particular, for MicroED, in addition to the standard direct methods.

### Completeness and observations over parameters

**Figure 3:**
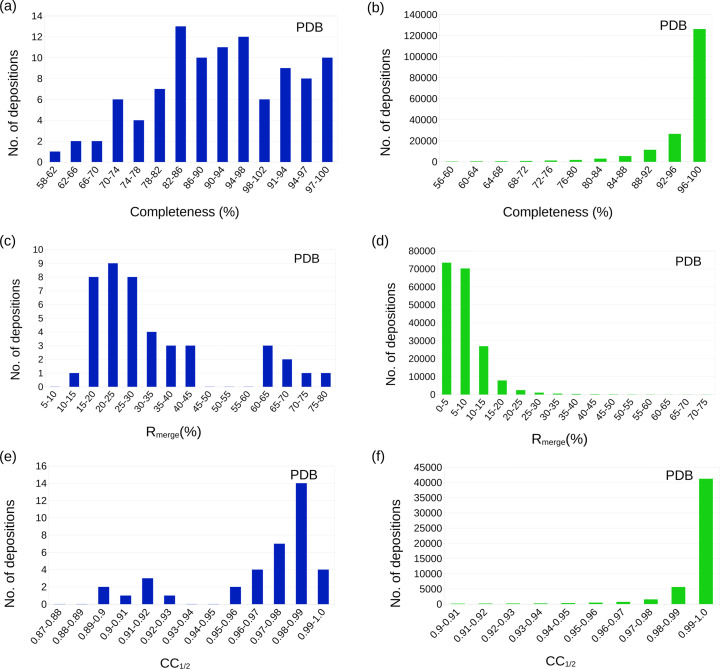
Statistics of the data collection parameters describing the data consistency of MicroED data (blue) and X-ray data (green). The number of PDB structures for bins of completeness are more distributed for the MicroED data (*a*) compared to the X-ray data (*b*). This can be inter­preted as a result of the limitations of the available tilting range in an electron microscope setup. In (*c*), the distribution of CC_1/2_ is plotted, revealing a typical value of above 0.85 with variations for the MicroED data and with more consistent values in the case of the X-ray data (*d*). The distribution of *R*_merge_ is plotted for MicroED data (*e*) and X-ray data (*f*) and is, together with CC_1/2_, related to the inter­nal consistency of the collected intensities. Larger differences can be seen in the MicroED data, however, the final refinements of the structures display similar results in terms of data quality.

A completeness of less than 100% can be a consequence of several reasons, such as limitations in the tilting range of the electron microscope stage, which in the case of MicroED is an intrinsic property of modern electron microscopes. Larger tilt angles may also result in higher absorption that could affect the *I*/σ(*I*) and the resolution at these angles. In contrast to expectations, as the distribution of the completeness for all the MicroED depositions in the PDB (MicroED) set plotted in Fig. 3 ▸(*a*) shows, most structures come from data with a com­pleteness of 70% or higher. It is common practice to merge data from several MicroED data collections. There are only a couple of macromolecular MicroED examples that exist where a significantly lower completeness was reported. While a lower completeness may result in difficulties in achieving a clear structure solution for both direct methods and MR approaches, a high completeness is key to a streamlined structure determination process and a reliable structure, as reflected by sound statistical metrics. The missing cone will have an adverse effect on the final potential map, resulting in uncertainties of the refined coordinates, as well as possessing a lower effective resolution in some direction from the sys­tem­atic lack of data. Considering these effects, a minimum completeness of 75% should become standard for a given resolution and structures determined with lower completeness should not be acceptable.

The eligibility of the refinement of small mol­ecules is sup­ported by the number of observed reflections and the number of parameters, as well as the number of restraints used in the refinement. While originally a ratio of 4 for the number of reflections to the number of parameters was considered enough, currently a ratio of 10 or more is deemed appropriate to support the refinement of the model as described in the *checkCIF* routine REFNR01. For comparison, the ‘refinement redundancy’ is calculated and presented in Table 2 ▸, and defined as:

#_ref_ = Redundancy in refinement = (No. of reflections + No. of restrictions)/No. of parameters [*I* > 2σ(*I*)]

As there is no consensus of an appropriate ratio with respect to MicroED data, we suggest the inclusion of the ratio, as well as the actual number of observations/restrictions and parameters refined, with the published structure. For the subset used from the CSD, the refinement redundancy ratios are 15.7 for the MicroED set and 16.4 for the electron diffraction excluded set. With both around 16, they are well above the reliable bar of 10. Not surprisingly, considering the similarity of the methods, we can confirm similar values for MicroED and X-ray crystallography.

### Merging *R* factors

To compensate for the limitations in the stage rotation range and weaker diffraction at higher tilt angles, data from several crystals will frequently be merged to increase the completeness for structure determination and the calculation of improved maps. Merging several data sets is common and works well for isomorphous data sets, but merging may be a non-trivial task for data sets not sufficiently isomorphous. Therefore, it has often become routine to identify groups of data sets that result in coherent structure factors, as determined by the correlation coefficients or any *R* factor related to merging, such as *R*_merge_, *R*_pim_, or *R*_meas_. The *R*_merge_ value can, for MicroED data, sometimes be substanti­ally higher than expected, between symmetry-related reflections within the data set, and between data from similar crystals there may be large variations. In the analyzed macromolecular groups displayed in Fig. 3 ▸(*c*), almost 70% of the data displays an *R*_merge_ value of 30% or lower, and most of the reported structures are found in the peak at 15–30% in *R*_merge_. With these numbers, which are also not unusual for X-ray data, especially for the high-resolution shells, the influence of non-isomorphism between crystals seems not to be a major concern. A smaller portion of the data resulted in an unusually high *R*_merge_ value between 50 and 77% (Lanza *et al.*, 2019 ▸; Takaba *et al.*, 2021 ▸; Martynowycz *et al.*, 2021 ▸; de la Cruz *et al.*, 2017 ▸). Nevertheless, even the data with rather high *R*_merge_ also resulted in well-refined structures and inter­pretable maps. In summary, based on the reported work, *R*_merge_ can be expected to fall in the region of 35% or lower, although it seems that a <!?up><!?tlsb><!?down>reasonable structure can occasionally be achieved for data that does not reach the expected levels. For small mol­ecule data, merging *R* values between data sets are often not reported.

### CC_1/2_

Karplus and Diederichs suggested in 2012 that a Pearson correlation coefficient between two subsets of the total data, CC_1/2_, is a better indicator of the quality and resolution of crystallographic data sets than more traditional measures (Karplus & Diederichs, 2012 ▸, 2015 ▸). Specifically, it was suggested that the CC_1/2_ could be analyzed in resolution bins to verify the extent of the diffracting pattern. It has been argued, as well as becoming a standard for many, that data in a resolution shell with a CC_1/2_ below 0.5 could still be used for structure refinement towards X-ray crystallographic data. For the subset of our analysis, the published MicroED structures report a high overall CC_1/2_ – better than 80% for all structures and better than 90% only with a few exceptions [Fig. 3 ▸(*e*)]. It is suggested that a CC_1/2_ of at least 90% for the overall data could be considered standard, while there may be less congruence in the value in the highest resolution bin.

### Residual *R* factors

**Figure 4:**
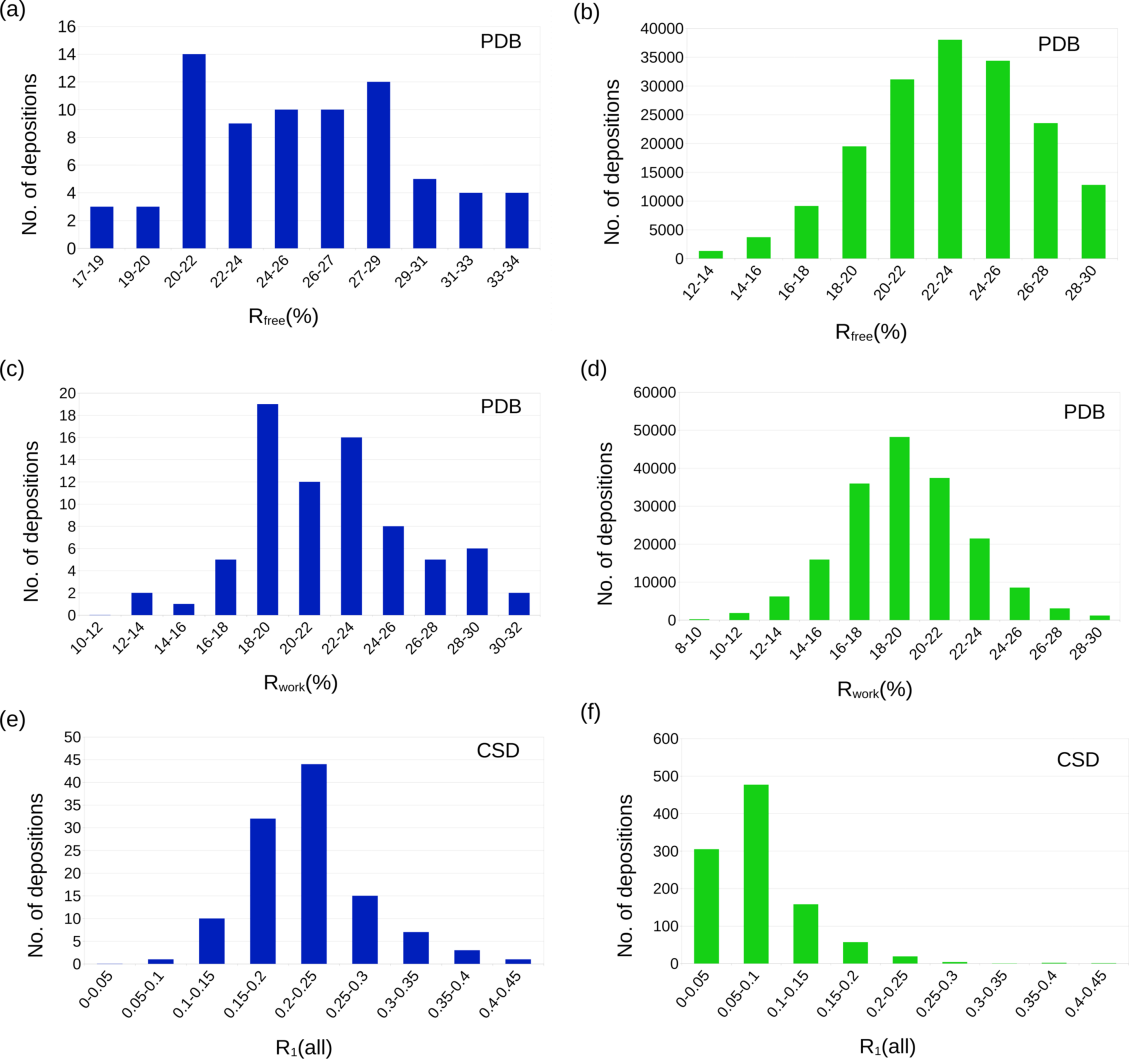
Statistics of the structure refinement parameters for macromolecular MicroED structures (blue) and X-ray structures (green) from the PDB (*a*)–(*d*) and the CSD (*e*)/(*f*). The distribution of both *R*_free_ (*a*) and *R*_work_ (*b*) are similar for the macromolecular MicroED and X-ray structures, with a slight shift towards higher values of about 2% for the MicroED data. Although inter­esting from the point of consistency within the PDB, the negligible difference also confirms the similar quality of the resulting structures for the two methods. The corresponding distribution of *R*_1_(all) in the CSD displays a larger difference of ∼0.15%. Small mol­ecule models are generally more complete than macromolecules, generating smaller *R* values; hence discrepancies between electron and non-electron data stand out better. Additionally, the larger number of reflections typically measured in macromolecular data might to some extent smear out the effect of dynamical scattering, which is affected by strong reflections and also varies between close reflections taking a similar trajectory through the crystal.

**Figure 5:**
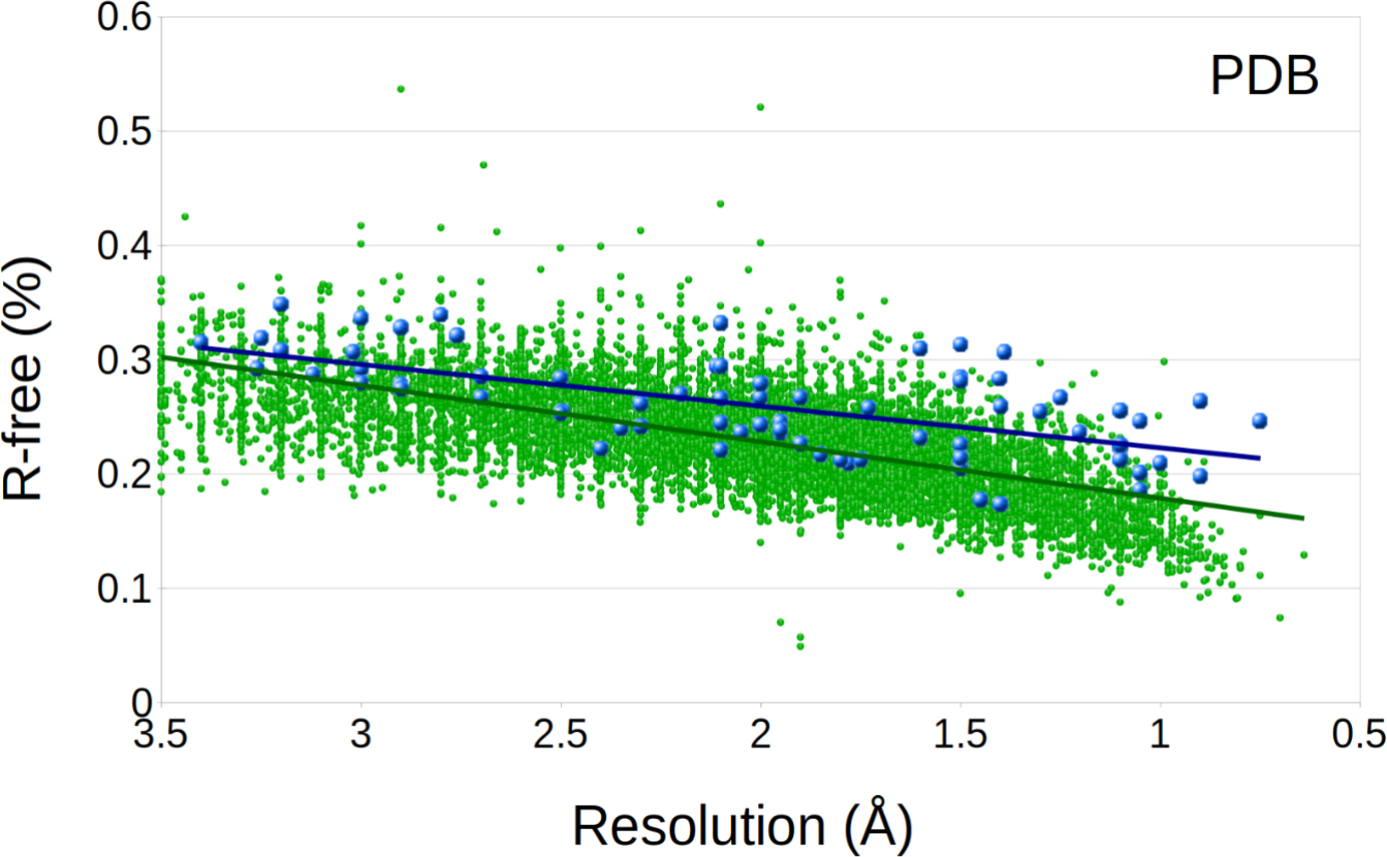
*R*_free_ plotted as a function of resolution, showing the macromolecular MicroED structures (blue points) and X-ray structures (green points). The trend lines are shown for the distribution of the PDB MicroED and X-ray data (blue and green lines, respectively). Although the trend lines for the MicroED and X-ray structures follow each other closely, *R*_free_ tends to be somewhat greater, in particular for the higher resolution structures. For visibility, 2% of the X-ray data was randomly selected for presentation.

It has been commonly anti­cipated that many of the structures determined by electron diffraction display residual *R* factors (*R*_1_, *R*_work_, and *R*_free_ for small mol­ecules and macromolecule refinement, respectively) that tend to be higher for MicroED data in comparison with X-ray data. As the distribution of the refined *R*_free_ for all X-ray structures in the PDB shows (Fig. 4 ▸), most deposited structures refine to an *R*_free_ value in the range 18–28%. The corresponding plot for MicroED indicates that a similar peak can be found at about 20–30% in *R*_free_, only about 2% higher in general. Similarly, the distribution of the *R*_work_ value displays a peak at 16–22% for the X-ray structures and a corresponding peak only slightly higher than for MicroED, at 18–24% in *R*_work_. While MicroED results in slightly higher *R* values, certain standards should be appropriate. Almost all MicroED structures, regardless of resolution, display *R*_work_ values less than 30% and for *R*_free_ less than 35%. As these numbers have been consistent in our laboratories, we believe they could serve as a standard goal for other MicroED studies. Plotted as a trend of the resolution (Figs. 5 ▸ and 6 ▸), the *R* values are generally improved for structures refined to a higher resolution, and structures with a resolution of 1.5 Å or better are generally found to achieve an *R*_work_ value of 25%. The trends of a lower *R*_free_ value at higher resolution can be understood as a better agreement of the model as the data becomes more well determined. Overall, the gap between X-ray and MicroED structures in terms of *R*_work_ and *R*_free_ is generally a few percent. In the small mol­ecule CIF files, the *R*_1_(all) is the conventional *R* factor within the given resolution inter­val for all data. The MicroED mean value of 0.23 for *R*_1_(all) is greater than similar values for non-MicroED data of 0.08. As small mol­ecule structural models typically include all available atoms, the discrepancy points to differences in the structure factors or structure factor calculations. As with the resolution (see *Conclusions*), both *R*_free_ and *R*_1_(all) have a wider distribution, which may be a result of sample selection, as discussed below. It may also be contributed to by differences in the structure factors, such as residuals of dynamical scattering and a comparably higher degree of inelastic background scattering in the MicroED data. In summary, there is no reason to believe that the difference in the residuals is related to not being able to model part of the structure; the models resulting from MicroED seem to contain a similar degree of detail as with X-ray-generated data. We anti­cipate that the slightly higher *R* values result from properties in the data, which as a result may contribute to some higher level of noise in the potential maps, but it is not related to properties of the final structure. An accurately refined model should not be of any concern for most applications.

### Goodness-of-fit (GooF)

The goodness-of-fit parameter takes the difference in structure factors and the number of observed reflections into account, and also the number of parameters used. At the end of a refinement, the GooF is expected to approach 1 and is mostly used for small mol­ecules. The fact that the mean value of 1.06 is closer to the ideal value of 1.0 for the CSD (Non-ED) group than the mean value of 1.7 for the CSD (MicroED) data may be for reasons similar to the differences in the structure factors, as discussed in previous sections. As with the resolution, the values for MicroED data contribute to a larger variety, with several structures reaching higher values.

### Atomic displacement parameters

The term Atomic Displacement Parameter or Anisotropic Displacement Parameter (ADP) has unfortunately been used in an inconsistent way historically. In the macromolecular world, it is often meant to refer to the *B* factor, while in the small mol­ecule literature it is referring to the metric tensor *U_ij_*. Both have the same dimension of Å^2^ describing the same phenomenon and are related by *B* = 8π^2^*U*. The *B* factor was originally referred to as the temperature factor or the Debye–Waller factor and was used to model the attenuation of diffraction intensities due to thermal motion, but later ADPs also became a way to model atom displacement and conformational or crystal disorder with a similar influence on the diffraction intensities. ADPs therefore provide a wide range of information, such as atomic motion, which are usually short distances, protein conformation disorder, as well as protein structure dynamics, which are typically large-scale movements. *B* factor values that are too large, for instance more than 100 Å^2^, are generally not considered to have any physical meaning (Carugo, 2018*a* ▸, 2018*b* ▸). Instead, their usage in structure refinement may lead to overfitting or mask an error of the choice of atom type, in particular for non-covalently bound atoms. Very high ADPs can sometimes be a motivation to instead use more descriptive attributes, such as a lower occupancy for a group of atoms, where alternative positions would be a better description.

The overall *B* is correlated with the resolution and within a non-redundant subset of the PDB it is found that:

*B* = 9*resolution^2^ (Å^2^)

For instance, a model determined to a high resolution of 1 Å has an overall *B* closer to 10 Å^2^, but for medium or lower resolution structures, higher *B* factors are common. In general, the overall *B* falls between 10 and 30 Å^2^ for most structures, while an *B* of lower than 10 Å^2^ or substanti­ally higher than 50 Å^2^ is less frequent. Instead, these less likely overall ADPs may indicate a problem with the model (or data), although exceptions do exist. An unreasonable ADP can mask an atom at the wrong position and it is important to confirm for any atom in doubt that its position is reasonable in the structure. During model building and refinement, it is useful to confirm a deviating ADP by checking the closest environment, such as surrounding charged or hydro­phobic atoms, coordination number, and type of coordinating atoms.

**Figure 7:**
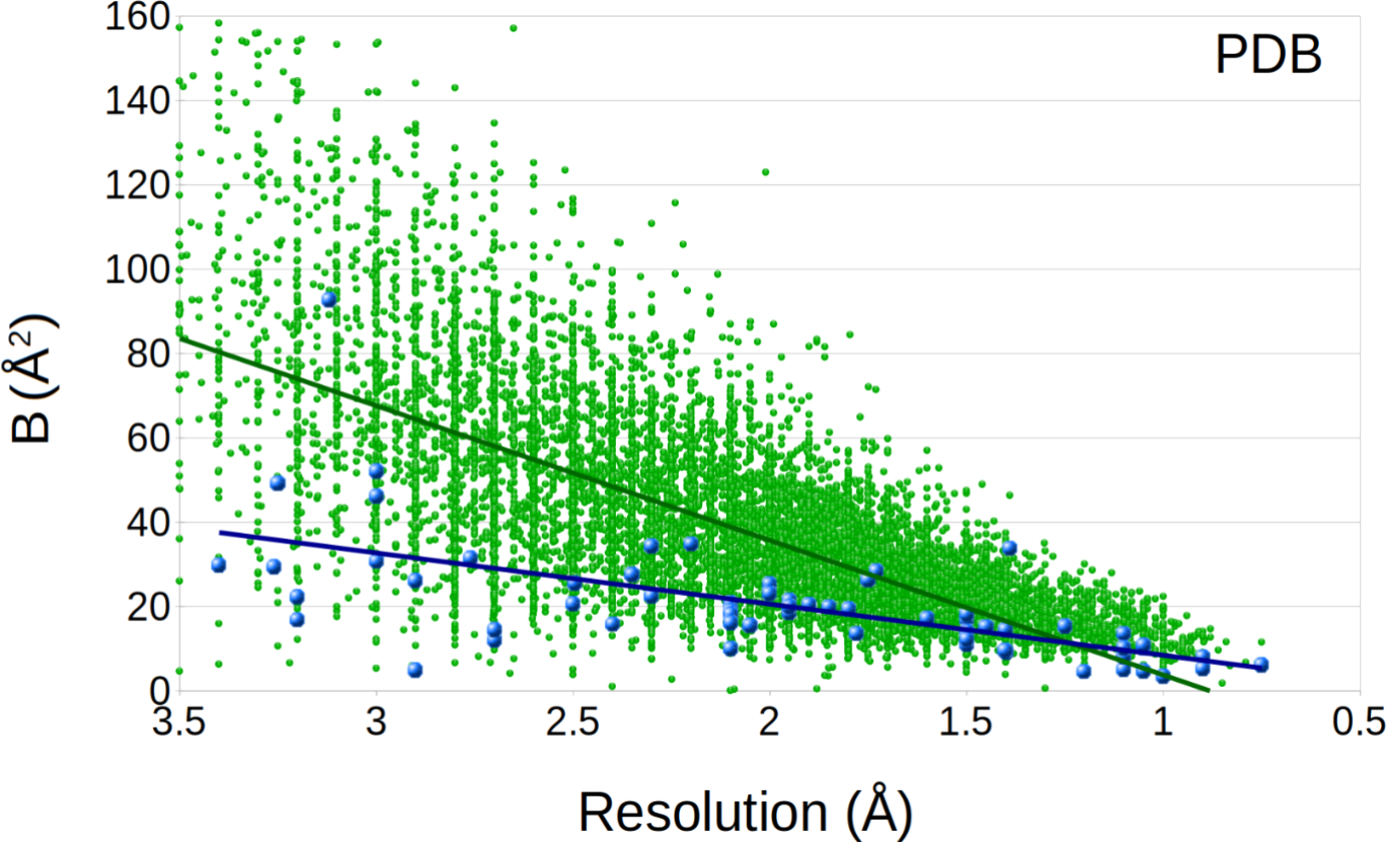
Overall *B* factors plotted as a function of resolution, showing the MicroED structures (blue points) and the X-ray structures (green points). The trend lines are shown for the distribution of the MicroED and X-ray data (blue and green lines, respectively). The overall *B* factor is substanti­ally lower for the MicroED structures, in particular at lower resolution. This may partially reflect the larger variation of structures solved using X-ray, but also demonstrates the high quality of the atomic coordinates in the refinement of the MicroED structures. For visibility, 2% of the X-ray data was randomly selected for presentation.

In an analysis of the ADPs of protein structures, Carugo observed that for X-ray structures, a resolution of 1.5 Å or better resulted in average *B* factors of about 25 and only at lower resolution did they tend to increase. As the resolution improves, the *B* factor decreases and its extrapolation would inter­sect with the resolution axis at about 0.5 Å. The plot of the model *B* factors *versus* the resolution of published MicroED structures shows an almost identical behavior: almost all mean temperature factors are below 20 Å^2^ at a resolution of 1.5 Å and the extrapolated tendency would inter­sect the resolution axis at around 0.5 Å or better (Fig. 7 ▸). The only noticeable difference is that the *B* factors are some­what lower for the MicroED subset. However, given the limited number of MicroED data sets in the comparison, it is difficult to speculate whether this is significant or if there are any reasons for the slightly lower *B* factor in the MicroED subset. The *B* factors of the structures in the PDB are far more distributed, some reaching very high values, whereas the numbers converge more as the resolution get better.

### Summary of quality indicators

Despite efforts to prepare comparative groups of structures for MicroED and Non-MicroED structures, there will be differences from factors outside of this scope. Several structures determined by MicroED are targets used as test samples for evaluating the method and therefore are expected to form well-behaving crystals, while many others represent a group of structures that have been unusually difficult to analyze using X-ray crystallography and where crystal growth was likely very limited, calling for the use of MicroED for these samples. It is probable that both the well-behaved and the limited crystal growth samples are reflected in the nanocrystals used in MicroED. A likely outcome could be the drawn-out nature of the statistical analysis. While MicroED also uses software tuned for X-ray crystallography, it is possible that MicroED data analysis workflows are less homogeneous compared with X-ray data, thus leading to a larger statistical range of refined models. As a main observation, it is illustrative to note that the parameters *R*_merge_ and CC_1/2_ are the only parameters in this analysis where clearer differences between MicroED and X-ray data can be discerned for structures in the PDB. These parameters are related to the inter­nal consistency of the measured intensities and describe the data before merging of the intensities and multiple data sets. The quality of the final structures, after merging of data and refinement of the structures, are better addressed by parameters from the refinement and structure evaluation. The take-away of these frequent comparisons to X-ray structures is that, while the statistical analysis of MicroED data may seem to be a matter of concern at the data collection stage, the quality of the resulting structures after data merging and refinement compares very well between MicroED and X-ray crystallography – as would be expected from substanti­ally related techniques. For instance, the resolution ends up with identical peaks for the most frequent resolution inter­vals, and the refinement *R* factors are overall similar and differ by just a couple of percent. While the resolution is strongly associated with the properties of the crystals, the *R*_merge_ and CC_1/2_ values are directly related to the inter­nal consistency of the data, which may depend on the data collection setup using an electron microscope, as well as the inter­action of the electron beam with the crystals. The refinement residuals on the other hand are related to the data only after merging. Agreement between the refinement residuals for MicroED and X-ray data reflects visually comparable outcomes of the two methods for macromolecular refinement and a small but consistent difference in the case of small mol­ecule refinement, which may be minimized taking dynamical scattering into account during refinement. This observation, however, neglects the differences in the sizes of the crystals. Overall, the resolution of a data collection using MicroED can be comparable to the resolution from data from X-ray crystallography data collection even though using crystals orders of magnitude smaller.

## Conclusions

More than a decade after the first MicroED structure was deposited and the method unveiled, there are more than a hundred macromolecular and several hundred small mol­ecule structures deposited in the PDB and CSD, respectively. As the number of refined structures with their data become available to the community, there are lessons that can be learned with respect to the profiles and quality of MicroED structures, as well as best practices. Here we have summarized the lessons learned from these data and proposed gold standards to be implemented by the community. Generally, the resulting models and density maps from MicroED are of high quality and comparable in resolution to X-ray crystallography. At this stage, the biggest hurdle for an even wider dissemination of the MicroED technology to the scientific community is the lack of experience, infrastructure, and national facilities with robust dedicated instruments for the method. As the tools for MicroED improve, and more cost-effective automatic microscopes for MicroED are deployed, we expect the use of MicroED to continue its rapid growth.

## Figures and Tables

**Figure 6:**
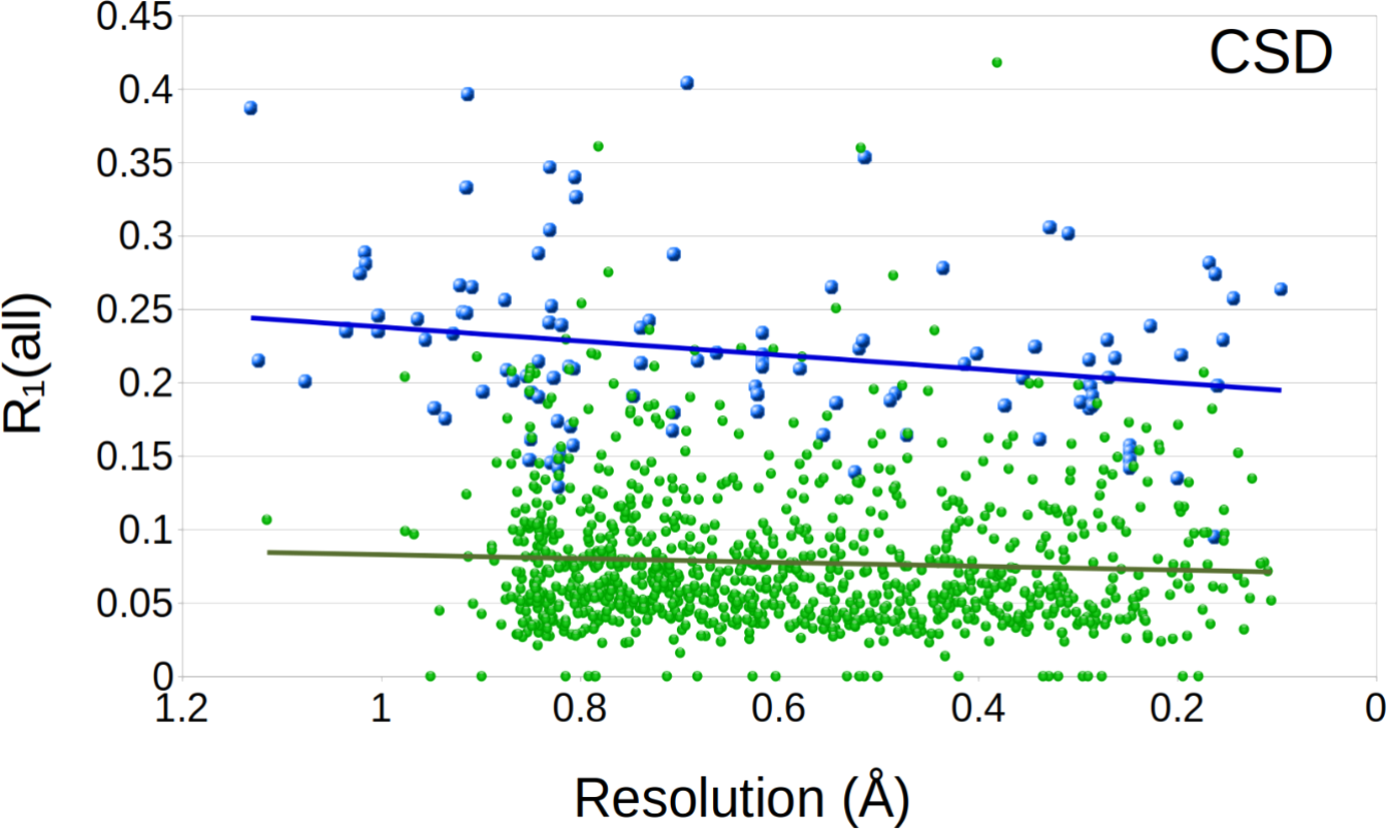
The *R*_1_(all) plotted as a function of resolution, showing the MicroED structures in the CSD group (MicroED set; blue points) and the comparative group (Non-ED group; green points). The trend lines are shown for the distribution of the MicroED and X-ray data (blue and green lines respectively).

**Figure 8:**
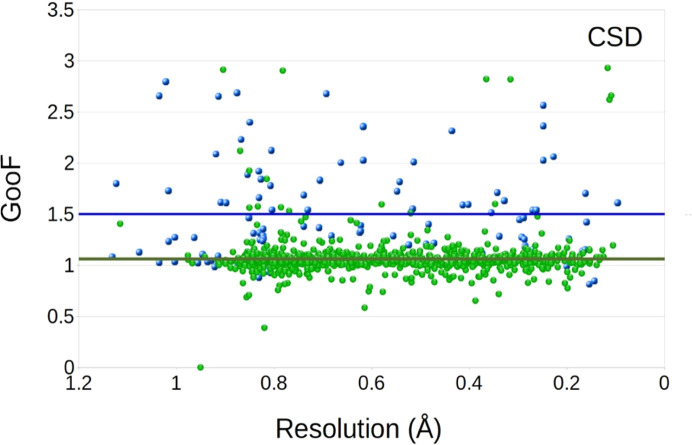
The distribution of goodness-of-fit (GooF) plotted as a function of resolution, showing the MicroED structures in the CSD group (MicroED set; blue points) and the comparative group (Non-ED group; green points). The lines with similar colors mark the mean values of the entire inter­val. Outliers are not included for visibility.

**Table 1:** Suggested data collection and structure refinement parameters to follow for publications of small mol­ecule MicroED structures shown together with their expected values

Parameter	Expected values	Mean values (MicroED)
Resolution (Å)	No limit	0.64
GooF	1	1.7
No. of reflections [*I* > 2σ(*I*)]	N/A	N/A
No. of parameters [*I* > 2σ(*I*)]	N/A	N/A
No. of restraints applied	N/A	N/A
Redundancy in refinement*	>10	15.7
*R*_1_(all) (*F*)	<0.35	0.23

Note: (*) #ref = (Redundancy in refinement) = (No. of reflections + No. of restraints)/No. of parameters [*I* > 2σ(*I*)]

**Table 2:** Statistics of the data collection and structure refinement of macromolecular data proposed as being required for every macromolecular MicroED study Although there is principally no difference between data for larger and smaller unit cells, it has become custom to use different sets of statistical parameters or with a different name for the data collection and refinement of small mol­ecules and macromolecules. We propose that the statistics of the data collection and structure refinement of macromolecular data, as presented in this table, in congruence with the profiles visualized in Figs. 2 ▸–5 ▸ ▸ ▸ and 7 ▸, should be required for every macromolecular MicroED study. Similarly, we suggest the inclusion of the statistics in Table 1 ▸, with the expected profiles of Figs. 6 ▸–8 ▸, for small-mol­ecule studies.

Parameter	Expected values	Mean values
Resolution (Å)	N/A	1.8
Completeness (%)	>70	85
Redundancy	No limit	4.2
*R*_merge_ (*I*)	<0.35	0.29
CC_1/2_	>0.9	0.97
*R*_free_	<∼0.30	0.25
<*R*_free_> – <*R*_work_>	<0.05	0.04
<*B*> (Å^2^)	<∼25	20

## References

[bb1] Andrusenko, I., Krysiak, Y., Mugnaioli, E., Gorelik, T. E., Nihtianova, D. & Kolb, U. (2015). *Acta Cryst.* B**71**, 349–357. 10.1107/S2052520615007994 26027011

[bb2] Battye, T. G. G., Kontogiannis, L., Johnson, O., Powell, H. R. & Leslie, A. G. W. (2011). *Acta Cryst.* D**67**, 271–281. 10.1107/S0907444910048675 PMC3069742 21460445

[bb3] Berman, H. M., Westbrook, J., Feng, Z., Gilliland, G., Bhat, T. N., Weissig, H., Shindyalov, I. N. & Bourne, P. E. (2000). *Nucleic Acids Res.* **28**, 235–242. 10.1093/nar/28.1.235 PMC102472 10592235

[bb4] Brázda, P., Klementová, M., Krysiak, Y. & Palatinus, L. (2022). *IUCrJ*, **9**, 735–755. 10.1107/S2052252522007904 PMC9634609 36381142

[bb5] Brink, J. & Chiu, W. (1994). *J. Struct. Biol.* **113**, 23–34. 10.1006/jsbi.1994.1029 7880650

[bb6] Bruhn, J. F., Scapin, G., Cheng, A., Mercado, B. Q., Waterman, D. G., Ganesh, T., Dallakyan, S., Read, B. N., Nieusma, T., Lucier, K. W., Mayer, M. L., Chiang, N. J., Poweleit, N., McGilvray, P. T., Wilson, T. S., Mashore, M., Hennessy, C., Thomson, S., Wang, B., Potter, C. S. & Carragher, B. (2021). *Front. Mol. Biosci.* **8**, https://doi.org/10.3389/fmolb.2021.648603. 10.3389/fmolb.2021.648603 PMC8313502 34327213

[bb7] Bruno, I. J., Cole, J. C., Edgington, P. R., Kessler, M., Macrae, C. F., McCabe, P., Pearson, J. & Taylor, R. (2002). *Acta Cryst.* B**58**, 389–397. 10.1107/s0108768102003324 12037360

[bb8] Bu, G., Danelius, E., Wieske, L. H. E. & Gonen, T. (2024). *Adv. Biol.* **8**, 2300570. 10.1002/adbi.202300570 PMC11090733 38381052

[bb9] Burla, M. C., Caliandro, R., Carrozzini, B., Cascarano, G. L., Cuocci, C., Giacovazzo, C., Mallamo, M., Mazzone, A. & Polidori, G. (2015). *J. Appl. Cryst.* **48**, 306–309.

[bb10] Carugo, O. (2018*a*). *Amino Acids*, **50**, 775–786. 10.1007/s00726-018-2574-y 29752562

[bb11] Carugo, O. (2018*b*). *BMC Bioinformatics*, **19**, 61. 10.1186/s12859-018-2083-8 PMC5824579 29471780

[bb12] Clabbers, M. T. B., Gruene, T., Parkhurst, J. M., Abrahams, J. P. & Waterman, D. G. (2018). *Acta Cryst.* D**74**, 506–518. 10.1107/S2059798318007726 PMC6096487 29872002

[bb13] Clabbers, M. T. B., Hattne, J., Martynowycz, M. W. & Gonen, T. (2025). *Nat. Commun.* **16**, 2247. 10.1038/s41467-025-57425-1 PMC11885823 40050283

[bb14] Clabbers, M. T. B., Holmes, S., Muusse, T. W., Vajjhala, P. R., Thygesen, S. J., Malde, A. K., Hunter, D. J. B., Croll, T. I., Flueckiger, L., Nanson, J. D., Rahaman, Md. H., Aquila, A., Hunter, M. S., Liang, M., Yoon, C. H., Zhao, J., Zatsepin, N. A., Abbey, B., Sierecki, E., Gambin, Y., Stacey, K. J., Darmanin, C., Kobe, B., Xu, H. & Ve, T. (2021). *Nat. Commun.* **12**, 2578. 10.1038/s41467-021-22590-6 PMC8110528 33972532

[bb15] Cowley, J. M. (1953). *Acta Cryst.* **6**, 516–521.

[bb16] Cowley, J. M. (1956). *Acta Cryst.* **9**, 391–396.

[bb17] Danelius, E., Bu, G., Wieske, H. & Gonen, T. (2023). *BioRxiv*, https://doi.org/10.1101/2023.07.31.551405.

[bb18] Danelius, E., Porter, N. J., Unge, J., Arnold, F. H. & Gonen, T. (2022). *BioRxiv*, https://doi.org/10.1101/2022.10.18.512604.

[bb19] de la Cruz, M. J., Hattne, J., Shi, D., Seidler, P., Rodriguez, J., Reyes, F. E., Sawaya, M. R., Cascio, D., Weiss, S. C., Kim, S. K., Hinck, C. S., Hinck, A. P., Calero, G., Eisenberg, D. & Gonen, T. (2017). *Nat. Methods*, **14**, 399–402. 10.1038/nmeth.4178 PMC5376236 28192420

[bb20] Dorset, D. L. (1999). *Microsc. Res. Tech.* **46**, 98–103. 10.1002/(SICI)1097-0029(19990715)46:2<98::AID-JEMT3>3.0.CO;2-G 10423555

[bb21] Dorset, D. L., Jap, B. K., Ho, M.-H. & Glaeser, R. M. (1979). *Acta Cryst.* A**35**, 1001–1009.

[bb22] Dorset, D. L. & Parsons, D. F. (1975*a*). *Acta Cryst.* A**31**, 210–215.

[bb23] Dorset, D. L. & Parsons, D. F. (1975*b*). *J. Appl. Cryst.* **8**, 12–14.

[bb24] Evans, P. R. & Murshudov, G. N. (2013). *Acta Cryst.* D**69**, 1204–1214. 10.1107/S0907444913000061 PMC3689523 23793146

[bb25] Fujiyoshi, Y. (1998). *Adv. Biophys.* **35**, 25–80. 10.1016/s0065-227x(98)90004-1 9949765

[bb26] Gillman, C., Bu, G., Danelius, E., Hattne, J., Nannenga, B. & Gonen, T. (2024). *BioRxiv*, https://doi.org/10.1101/2024.01.11.575283.

[bb27] Gillman, C., Patel, K., Unge, J. & Gonen, T. (2023). *BioRxiv*, https://doi.org/10.1101/2023.03.31.535166.

[bb28] Gonen, T. (2013). *Electron Crystallography of Soluble and Membrane Proteins: Methods and Protocols*, edited by I. Schmidt-Krey & Y. Cheng, pp. 153–169. Totowa, NJ: Humana Press.

[bb29] Gonen, T., Cheng, Y., Sliz, P., Hiroaki, Y., Fujiyoshi, Y., Harrison, S. C. & Walz, T. (2005). *Nature*, **438**, 633–638. 10.1038/nature04321 PMC1350984 16319884

[bb30] Gonen, T., Sliz, P., Kistler, J., Cheng, Y. & Walz, T. (2004). *Nature*, **429**, 193–197. 10.1038/nature02503 15141214

[bb31] Gorelik, T. E., Lukat, P., Kleeberg, C., Blankenfeldt, W. & Mueller, R. (2023). *Acta Cryst.* A**79**, 504–514. 10.1107/S2053273323008458 PMC10626656 37855135

[bb32] Grigorieff, N., Ceska, T. A., Downing, K. H., Baldwin, J. M. & Henderson, R. (1996). *J. Mol. Biol.* **259**, 393–421. 10.1006/jmbi.1996.0328 8676377

[bb33] Groom, C. R., Bruno, I. J., Lightfoot, M. P. & Ward, S. C. (2016). *Acta Cryst.* B**72**, 171–179. 10.1107/S2052520616003954 PMC4822653 27048719

[bb34] Hattne, J., Clabbers, M. T. B., Martynowycz, M. W. & Gonen, T. (2023). *BioRxiv*, https://doi.org/10.1101/2023.06.29.547123.

[bb35] Hattne, J., Reyes, F. E., Nannenga, B. L., Shi, D., de la Cruz, M. J., Leslie, A. G. W. & Gonen, T. (2015). *Acta Cryst.* A**71**, 353–360. 10.1107/S2053273315010669 PMC4487423 26131894

[bb36] Henderson, R. & Unwin, P. N. (1975). *Nature*, **257**, 28–32. 10.1038/257028a0 1161000

[bb37] Jeng, T.-W. & Chiu, W. (1984). *Ultramicroscopy*, **13**, 27–34. 10.1016/0304-3991(84)90054-8 6474598

[bb38] Jones, C. G., Martynowycz, M. W., Hattne, J., Fulton, T. J., Stoltz, B. M., Rodriguez, J. A., Nelson, H. M. & Gonen, T. (2018). *ACS Cent. Sci.* **4**, 1587–1592. 10.1021/acscentsci.8b00760 PMC6276044 30555912

[bb39] Jumper, J., Evans, R., Pritzel, A., Green, T., Figurnov, M., Ronneberger, O., Tunyasuvunakool, K., Bates, R., Žídek, A., Potapenko, A., Bridgland, A., Meyer, C., Kohl, S. A. A., Ballard, A. J., Cowie, A., Romera-Paredes, B., Nikolov, S., Jain, R., Adler, J., Back, T., Petersen, S., Reiman, D., Clancy, E., Zielinski, M., Steinegger, M., Pacholska, M., Berghammer, T., Bodenstein, S., Silver, D., Vinyals, O., Senior, A. W., Kavukcuoglu, K., Kohli, P. & Hassabis, D. (2021). *Nature*, **596**, 583–589. 10.1038/s41586-021-03819-2 PMC8371605 34265844

[bb40] Kabsch, W. (2010*a*). *Acta Cryst.* D**66**, 133–144. 10.1107/S0907444909047374 PMC2815666 20124693

[bb41] Kabsch, W. (2010*b*). *Acta Cryst.* D**66**, 125–132. 10.1107/S0907444909047337 PMC2815665 20124692

[bb42] Karothu, D. P., Alhaddad, Z., Göb, C. R., Schürmann, C. J., Bücker, R. & Naumov, P. (2023). *Angew. Chem. Int. Ed.* **62**, e202303761. 10.1002/anie.202303761 37071841

[bb43] Karplus, P. A. & Diederichs, K. (2012). *Science*, **336**, 1030–1033. 10.1126/science.1218231 PMC3457925 22628654

[bb44] Karplus, P. A. & Diederichs, K. (2015). *Curr. Opin. Struct. Biol.* **34**, 60–68. 10.1016/j.sbi.2015.07.003 PMC4684713 26209821

[bb45] Kim, L. J., Ohashi, M., Zhang, Z., Tan, D., Asay, M., Cascio, D., Rodriguez, J. A., Tang, Y. & Nelson, H. M. (2021*a*). *Nat. Chem. Biol.* **17**, 872–877. 10.1038/s41589-021-00834-2 PMC8447837 34312563

[bb46] Kim, L. J., Xue, M., Li, X., Xu, Z., Paulson, E., Mercado, B., Nelson, H. M. & Herzon, S. B. (2021*b*). *J. Am. Chem. Soc.* **143**, 6578–6585. 10.1021/jacs.1c01729 PMC8935351 33900077

[bb47] Kimura, Y., Vassylyev, D. G., Miyazawa, A., Kidera, A., Matsushima, M., Mitsuoka, K., Murata, K., Hirai, T. & Fujiyoshi, Y. (1997). *Nature*, **389**, 206–211. 10.1038/38323 9296502

[bb48] Kühlbrandt, W., Wang, D. N. & Fujiyoshi, Y. (1994). *Nature*, **367**, 614–621. 10.1038/367614a0 8107845

[bb49] Lanza, A., Margheritis, E., Mugnaioli, E., Cappello, V., Garau, G. & Gemmi, M. (2019). *IUCrJ*, **6**, 178–188. 10.1107/S2052252518017657 PMC6400191 30867915

[bb50] Liebschner, D., Afonine, P. V., Baker, M. L., Bunkóczi, G., Chen, V. B., Croll, T. I., Hintze, B., Hung, L.-W., Jain, S., McCoy, A. J., Moriarty, N. W., Oeffner, R. D., Poon, B. K., Prisant, M. G., Read, R. J., Richardson, J. S., Richardson, D. C., Sammito, M. D., Sobolev, O. V., Stockwell, D. H., Terwilliger, T. C., Urzhumtsev, A. G., Videau, L. L., Williams, C. J. & Adams, P. D. (2019). *Acta Cryst.* D**75**, 861–877. 10.1107/S2059798319011471 PMC6778852 31588918

[bb51] Lightowler, M., Li, S., Ou, X., Zou, X., Lu, M. & Xu, H. (2022). *Angew. Chem. Int. Ed.* **61**, e202114985. 10.1002/anie.202114985 PMC9306882 34902212

[bb52] Lin, J., Bu, G., Unge, J. & Gonen, T. (2024*a*). *BioRxiv*, https://doi.org/10.1101/2024.06.05.597682.

[bb53] Lin, J., Bu, G., Unge, J. & Gonen, T. (2024*b*). *BioRxiv*, https://doi.org/10.1101/2024.01.04.574265.

[bb54] Lin, J., Unge, J. & Gonen, T. (2023*a*). *BioRxiv*, https://doi.org/10.1101/2023.06.28.546957.

[bb55] Lin, J., Unge, J. & Gonen, T. (2023*b*). *BioRxiv*, https:/doi.org/10.1101/2023.09.05.556418.

[bb56] Martynowycz, M. W., Clabbers, M. T. B., Hattne, J. & Gonen, T. (2022). *Nat. Methods*, **19**, 724–729. 10.1038/s41592-022-01485-4 PMC9184278 35637302

[bb57] Martynowycz, M. W., Clabbers, M. T. B., Unge, J., Hattne, J. & Gonen, T. (2021). *Proc. Natl. Acad. Sci. USA*, **118**, e210884118. 10.1073/pnas.2108884118 PMC8670461 34873060

[bb58] Martynowycz, M. W., Zhao, W., Hattne, J., Jensen, G. J. & Gonen, T. (2019). *Structure*, **27**, 545–548. 10.1016/j.str.2018.12.003 PMC6476546 30661853

[bb59] Miller, J. E., Agdanowski, M. P., Dolinsky, J. L., Sawaya, M. R., Cascio, D., Rodriguez, J. A. & Yeates, T. O. (2024). *Acta Cryst.* D**80**, 270–278. 10.1107/S205979832400072X PMC10994174 38451205

[bb60] Murata, K., Mitsuoka, K., Hirai, T., Walz, T., Agre, P., Heymann, J. B., Engel, A. & Fujiyoshi, Y. (2000). *Nature*, **407**, 599–605. 10.1038/35036519 11034202

[bb61] Murshudov, G. N., Vagin, A. A. & Dodson, E. J. (1997). *Acta Cryst.* D**53**, 240–255. 10.1107/S0907444996012255 15299926

[bb62] Nannenga, B. L., Shi, D., Leslie, A. G. W. & Gonen, T. (2014). *Nat. Methods*, **11**, 927–930. 10.1038/nmeth.3043 PMC4149488 25086503

[bb63] Oleynikov, P., Hovmöller, S. & Zou, X. D. (2007). *Ultramicroscopy*, **107**, 523–533. 10.1016/j.ultramic.2006.04.032 17291687

[bb64] Otwinowski, Z. & Minor, W. (1997). *Methods in Enzymology*, Vol. 276, *Macromolecular Crystallography*, Part A, edited by C. W. Carter Jr & R. M. Sweet, pp. 307–326. New York: Academic Press. 10.1016/S0076-6879(97)76066-X 27754618

[bb65] Palatinus, L., Brázda, P., Jelínek, M., Hrdá, J., Steciuk, G. & Klementová, M. (2019). *Acta Cryst.* B**75**, 512–522. 10.1107/S2052520619007534 32830709

[bb66] Palatinus, L. & Chapuis, G. (2007). *J. Appl. Cryst.* **40**, 786–790.

[bb67] Parkhurst, J. M., Winter, G., Waterman, D. G., Fuentes-Montero, L., Gildea, R. J., Murshudov, G. N. & Evans, G. (2016). *J. Appl. Cryst.* **49**, 1912–1921. 10.1107/S1600576716013595 PMC5139990 27980508

[bb68] Porter, N. J., Danelius, E., Gonen, T. & Arnold, F. H. (2022). *J. Am. Chem. Soc.* **144**, 8892–8896. 10.1021/jacs.2c02723 PMC9205183 35561334

[bb69] Rajashankar, K. & Dauter, Z. (2014). *Structural Genomics and Drug Discovery: Methods and Protocols*, edited by W. F. Anderson, pp. 211–237. New York: Springer.

[bb70] Richards, L. S., Flores, M. D., Millán, C., Glynn, C., Zee, C.-T., Sawaya, M. R., Gallagher-Jones, M., Borges, R. J., Usón, I. & Rodriguez, J. A. (2021). *BioRxiv*, https://doi.org/10.1101/2021.09.13.459692.

[bb71] Sekharan, S., Liu, X., Yang, Z., Liu, X., Deng, L., Ruan, S., Abramov, Y., Sun, G., Li, S., Zhou, T., Shi, B., Zeng, Q., Zeng, Q., Chang, C., Jin, Y. & Shi, X. (2021). *RSC Adv.* **11**, 17408–17412. 10.1039/d1ra03100g PMC9033196 35479679

[bb72] Sheldrick, G. M. (1990). *Acta Cryst.* A**46**, 467–473.

[bb73] Sheldrick, G. M. (2010). *Acta Cryst.* D**66**, 479–485. 10.1107/S0907444909038360 PMC2852312 20383001

[bb74] Sheldrick, G. M. (2015). *Acta Cryst.* A**71**, 3–8.

[bb75] Shi, D., Lewis, M. R., Young, H. S. & Stokes, D. L. (1998). *J. Mol. Biol.* **284**, 1547–1564. 10.1006/jmbi.1998.2283 9878370

[bb76] Shi, D., Nannenga, B. L., Iadanza, M. G. & Gonen, T. (2013). *Elife*, **2**, e01345. 10.7554/eLife.01345 PMC3831942 24252878

[bb77] Takaba, K., Maki-Yonekura, S., Inoue, S., Hasegawa, T. & Yonekura, K. (2021). *Front. Mol. Biosci.* **7**, https://doi.org/10.3389/fmolb.2020.612226. 10.3389/fmolb.2020.612226 PMC7814344 33469549

[bb78] Unge, J., Lin, J., Weaver, S. J., Sae Her, A. & Gonen, T. (2024). *Adv. Sci.* **11**, 2400081. 10.1002/advs.202400081 PMC11187898 38647272

[bb79] Unwin, P. N. T. & Henderson, R. (1975). *J. Mol. Biol.* **94**, 425–440. 10.1016/0022-2836(75)90212-0 1236957

[bb80] Vainshtein, B. K. & Pinsker, Z. G. (1949). *J. Phys. Chem. SSSR*, **23**, 1058–1067.

[bb81] Vincent, R. & Midgley, P. A. (1994). *Ultramicroscopy*, **53**, 271–282.

[bb82] Xu, H., Lebrette, H., Clabbers, M. T. B., Zhao, J., Griese, J. J., Zou, X. & Högbom, M. (2019). *Sci. Adv.* **5**, eaax4621. 10.1126/sciadv.aax4621 PMC6685719 31457106

[bb83] Yokoo, H., Aoyama, Y., Matsumoto, T., Yamamoto, E., Uchiyama, N. & Demizu, Y. (2024). *Chem. Pharm. Bull.* **72**, 471–474. 10.1248/cpb.c23-00745 38749738

